# Symbiotic Effectivity of Dual and Tripartite Associations on Soybean (*Glycine max* L. Merr.) Cultivars Inoculated With *Bradyrhizobium japonicum* and AM Fungi

**DOI:** 10.3389/fpls.2018.01631

**Published:** 2018-11-13

**Authors:** Tünde Takács, Imre Cseresnyés, Ramóna Kovács, István Parádi, Bettina Kelemen, Tibor Szili-Kovács, Anna Füzy

**Affiliations:** ^1^Institute for Soil Sciences and Agricultural Chemistry, Centre for Agricultural Research, Hungarian Academy of Sciences, Budapest, Hungary; ^2^Department of Plant Physiology and Molecular Plant Biology, Institute of Biology, Eötvös Loránd University, Budapest, Hungary

**Keywords:** arbuscular mycorrhizal fungi, *Bradyrhizobium japonicum*, drought stress, functional diversity, root electrical capacitance, soybean, symbiotic compatibility

## Abstract

Soybean (*Glycine max* L. Merr.) is regarded worldwide as indisputably one of the most important crops for human food and animal feed. The presence of symbiotic bacteria and fungi is essential for soybean breeding, especially in low-input agricultural systems. Research on the cooperation between different microbial symbionts is a key to understanding how the health and productivity of the plant is supported. The symbiotic effectivity of dual and tripartite symbiotic agents was investigated in two pot experiments on different soybean cultivars with special regard to compatibility. In the Selection experiment, two out of sixteen soybean cultivars (Aliz, Emese) were chosen on the basis of their drought tolerance and used in all the other investigations. In the Compatibility experiment, the compatible coupling of symbiotic partners was selected based on the efficiency of single and co-inoculation with two *Bradyrhizobium japonicum* strains and two commercial arbuscular mycorrhizal fungal (AMF) products. Significant differences were found in the infectivity and effectivity of the microsymbionts. The rhizobial and AMF inoculation generally improved plant production, photosynthetic efficiency and root activity, but this effect depended on the type of symbiotic assotiation. Despite the low infectivity of AMF, inocula containing fungi were more beneficial than those containing only rhizobia. In the Drought Stress (DS) experiment, co-inoculated and control plants were grown in chernozem soil originating from organic farms. Emese was more resistant to drought stress than Aliz and produced a bigger root system. Under DS, the growth parameters of both microbially inoculated cultivars were better than that of control, proving that even drought tolerant genotypes can strengthen their endurance due to inoculation with AMF and nitrogen fixing bacteria. Root electrical capacitance (C_R_) showed a highly significant linear correlation with root and shoot dry mass and leaf area. The same root biomass was associated with higher C_R_ in inoculated hosts. As C_R_ method detects the absorptive surface increasing due to inoculation, it may be used to check the efficiency of the microbial treatment.

## Introduction

Soybean (*Glycine max* L. Merr.) is one of the most widely cultivated crops throughout the world under various climates. Future scenarios show that soybean production will expand by 30% for the coming decade (OECD-FAO, 2018), so optimizing the cultivation of this crop has important economic and political implications.

Achieving this purpose, however, is complicated by the extreme weather conditions caused by global climate change. Crop growth and yield are impaired by both abiotic and biotic stress conditions, of which drought has been identified as the most important factor in limiting the productivity of grain legumes (Farooq et al., 2016). Drought sensitivity coupled with high nutrient demand may seriously restrict soybean yields due to weak development and poor competitiveness against weeds. Therefore, the development of resistant cultivars and site-specific selection from the available genetic resources should be major objectives in any breeding program. The significance of nitrogen-fixing bacteria and arbuscular mycorrhizal fungal (AMF) symbionts is indubitable not only in soybean host nutrition, but also in the alleviation of plant stress caused by adverse soil conditions (Ku et al., 2013).

Nitrogen (N) and phosphorus (P) are critical limiting elements for crop growth (Miller and Cramer, 2004; Menge et al., 2012). Legumes require nitrogen-fixing rhizobial symbiotic partners, mostly *Bradyrhizobium japonicum* bacteria, to achieve their maximum yield potential. Soils in areas where soybean is not native usually lack these bacteria, e.g., in. in Europe (Albareda *et al.*, 2009), so the microbial inoculation of soybean is essential to provide adequate nitrogen supplies and maximum yields both in conventional agricultural practices and more especially in organic farming.

Leguminous plants are highly dependent also on mycorrhizal fungi (Muleta, 2010), symbionts that can resolve the problem of phosphorus limitation. AM fungi live in a mutualistic symbiosis with 80–90% of terrestrial plants and form the most ancient and prevalent type of mycorrhizae (Brundrett, 2009). The improved productivity of AM plants and the physiological and biochemical changes caused by AM can result in greater stress resistance in the host (Cameron et al., 2013; Rapparini and Peñuelas, 2014; Domokos et al., 2018). The extraradical hyphal network of AMF provides more effective water and nutrient (especially plant-unavailable phosphorus) uptake for the host plants (Marschner, 1997; Hodge and Storer, 2015; Wahbi et al., 2016; Wang et al., 2016; Balog et al., 2017). Furthermore, the host plants benefit from the special composition of the microbial community in the mycorrhizosphere (Barea, 1997; Artusson et al., 2006).

Recent studies confirm that there is a common genetic basis for plant root endosymbioses with both rhizobia and AM fungi (Denison and Kiers, 2011; Ossler et al., 2015). AMF colonization has an influence on the development and function of rhizobial nodules and vice versa, but the multiple mutualistic effect on the host participating in the rhizobia–AM fungi–legume interaction is very fertilizer-dependent. Numerous publications reported that the co-inoculation of legumes with AMF and rhizobial strains resulted in greater benefits for the plants and symbionts alike, due to a synergistic effect (Bethlenfalvay et al., 1987; Biró et al., 2000; Meghvansi et al., 2008; Wang et al., 2011). Neutral or negative responses to co-inoculation indicated that the advantage of tripartite symbiotic associations depended greatly on the compatibility and susceptibility of the partners (Lisette et al., 2003; Wang et al., 2011). As a principal effect, AM fungi improve nodulation in the legume host by enhancing phosphorus uptake (Ossler et al., 2015). In addition, AMF root colonization leads to changes in the nitrogen transfer, microelement uptake and phytohormone production of the plants, which play an important role in nodulation and nitrogen fixation (Behie and Bidochka, 2014). The multi-factorial evaluation of tripartite functional diversity could provide a theoretical basis for optimizing the application of selected biofertilizers for soybean production.

The varying efficiency of biological nitrogen fixation and fungal colonization both show that the compatibility of the mutualistic partners depends on their genotype. Due to intra- and interspecific variability, several possible partners are in the race for the formation of symbiosis, but the actual outcome of colonization is also influenced by the environmental conditions (Johnson et al., 1997; Kiers et al., 2002; Herrera-Peraza et al., 2011). In general, associations of rhizobia and host plants have narrow specificity during nodule development (Fauvart and Michiels, 2008). The association between the bacteria and the soybean may be host-specific, to such a degree that some rhizobial species nodulate plants only in a certain genus (Neves and Rumjanek, 1997; Appunuu et al., 2008). The variability of bradyrhizobial effectiveness on different soybean varieties was reported by Okereke et al. (2001). In contrast, the AMF-host relationship is not strictly specific; the high intra- and interspecific variability of AM fungi creates great functional diversity (Jakobsen et al., 1992; Munkvold et al., 2004; Cavagnaro et al., 2005).

The great functional diversity and non-host specific association of AM fungi give a chance to produce biofertilizers that can establish fungi–host combinations under diverse environmental conditions. Despite the increasing attention paid to AMF as an advantageous symbiotic partner, a number of difficulties have so far prevented the large-scale application of AMF inoculation (Cely et al., 2016). In low-input organic systems, compatible plant–fungus–rhizobium associations may play a more prominent and critical role in the optimal nutrition of the host plants than in conventional agricultural systems (Gosling et al., 2006; Bhardwaj et al., 2014; Schneider et al., 2015). In organic farming, choosing a suitable cultivar is essential to avoid the damaging effects of environmental stress. The metabolism of drought-tolerant and non-tolerant cultivars has been compared (Purcell et al., 2000; Silvente et al., 2012), but few data are available on cultivars with similar tolerance and on the role of symbionts in endurance.

Well known, that there are differences in the drought tolerance of the registered soybean cultivars (Bouslama and Schapaugh, 1984; Cseresnyés et al., 2016). It is assumed, that even drought tolerant soybeans benefit from inoculation, but because of the various compatibility cultivars show different responses to microbial treatments. For the rational use of commercial inocula, their efficiency should be checked before application in local soils under different environmental conditions. It is also assumed that AMF will add to drought tolerance more than rhizobium considering the active surfaces. The main objective of the experiments was to show the significance of the compatibility of symbiotic partners in a soybean tripartite inoculation and provide the theoretical basis for the application of commercial products in the soybean field production. To asses the effect of symbioses, it is practical to measure multiple growth, physiological and morphological parameters related to all the partners. In the present study, commercial products containing *Bradyrhizobium japonicum* and AM fungal inocula were tested for symbiotic effectivity on soybean in different growth media. The host susceptibility to inoculation and the impact of the soil biotic context (belowground interactions) were investigated to reveal their effect on the compatibility of the symbiotic partners. The functionality of the symbiotic partners was also checked in relation to genotypic differences in the soybean cultivars under drought stress.

## Materials and Methods

### The Sequence of the Experiments

In the Selection experiment 16 soybean (*Glycine max* L. Merr.) genotypes of diverse origin (Table 1) were evaluated for drought tolerance by means of polyethylene glycol (PEG) – induced drought stress. The Compatibility pot experiment was than performed to test two bradyrhizobia and two AM fungi (alone and in pairs) by inoculating two selected soybean cultivars grown in pumice to identify the most effective combinations of symbiotic partners. Finally, the Drought Stress pot experiment was performed to study symbiotic efficiency under optimal water supplies and water deficit in soil from organic farms. The test plants, methods of plant cultivation and tests for symbiotic effectiveness were the same in both pot experiments.

**Table 1:** Characteristics of soybean cultivars tested in the germination experiment.

Cultivar (Year of state registration)	Maturity group	1000-grain weight^a^ (g)	${\mathrm{GSI}}_{6}^{b}$ (%)
BAGERA (2007, Switzerland)	[00]	148.7 (7.0)	29.4 (11.4)
ES MENTOR (2011, France)	[00]	189.0 (3.5)	13.3 (11.2)
ALIZ (2007, Hungary)	[0]	182.2 (5.2)	60.3 (4.5)
JOHANNA (2011, Hungary)	[0]	208.3 (6.2)	19.2 (11.6)
MARTINA (2006, Hungary)	[0]	202.3 (5.2)	23.8 (10.2)
SPLENDOR (2006, EU)	[0]	180.0 (2.7)	0.0
ES GLADIÁTOR (2014, France)	[0-I]	180.9 (3.0)	10.77 (6.11)
EMESE (2006, Hungary)	[I]	176.4 (3.3)	54.7 (10.8)
ISIDOR (2005, EU)	[I]	219.0 (5.6)	17.9 (15.9)
PANNÓNIA KINCSE (2008, Hungary)	[I]	130.1 (3.3)	1.0 (2.0)
SPONSOR (2005, France)	[I]	156.7 (3.2)	43.9 (10.7)
ZELMA (2008, Hungary)	[I]	168.7 (7.7)	29.5 (9.6)
GROWPRO (United States)	[II]	223.1 (3.3)	11.8 (7.9)
HIPRO 15/MN 1505 (2010, United States)	[II]	198.1 (4.6)	17.1 (8.0)
ROYALPRO (2006, United States)	[II]	247.5 (4.1)	11.0 (1.0)
PRESTOPRO (United States)	[II]	117.7 (1.5)	27.4 (12.0)


*^*a*^Based upon weighing 5 × 100 seeds; standard deviations are given in brackets. ^*b*^GSI_*6*,_ Germination stress index after 6 days of germination.*

### Selection Experiment

The seeds were selected for size homogeneity, surface-sterilized for 0.5 min in 70% (v/v) ethyl alcohol, rinsed and soaked in sterile distilled water. Seed germination was studied in distilled water (control) and in a 25 w/w% solution of polyethylene glycol (PEG, Karbowax 6000 Fluka AG) generating an osmotic potential of -0.82 MPa (Michel and Kaufmann, 1973). For each genotype, four replicates (*n* = 4) of 25 seeds were placed in Petri dish (Ø 9 cm) on a filter paper that covered a piece of cotton wool (4 g). The dishes were filled with 40 cm^3^ of distilled water or PEG solution. The seeds were incubated in a dark, temperature-controlled chamber at 25 ± 1°C. Seeds were considered germinated when the radicle had reached at least 2 mm in length. The number of germinated seeds was counted daily. After 6 days, the GSI_6_ was calculated according to Bouslama and Schapaugh (1984). In the Compatibility and Drought Stress experiments the two cultivars with the highest GSI_6_ (Aliz and Emese, indeterminate growth habit, maturity group 0) were used as host for the microsymbionts.

### Compatibility Experiment

The Aliz and Emese cultivars were investigated for their compatibility with AM fungi and rhizobial inoculants. Biomass production (shoot and root dry weight; SDW and RDW), LA, photochemical efficiency (F_v_/F_m_) and root electrical capacitance (C_R_) as an indicator of root system activity, were measured in the early developmental stages. Root colonization with AMF, nodulating parameters and acetylene reduction were tested to estimate the functionality of the symbiotic partners.

AMF inoculation with a commercial product (either F_1_ or F_2_) and rhizobial inoculation with a commercially available soybean inoculum (R_1_) or a *Bradyrhizobium japonicum* strain (R_2_) were applied as microbial treatments. Control (C) plants were not inoculated.

F_1_ is a 1:1 mixed inoculum of *Rhizophagus intraradices* (syn. *Glomus intraradices*) and *Funneliformis mosseae* (syn. *Glomus mosseae*) with 50 spores g^-1^. It is characterized by high organic matter content (20%), pH of 6.5–7.0, ammonium lactate (AL)-K_2_O 8364 mg kg^-1^, AL-P_2_O_5_ 4987 mg kg^-1^ and 10^7^ colony-forming units of rhizosphere bacteria (CFU g^-1^) in microgranular form (1–2 mm). F_2_ is an infective propagule mixture of six AMF strains, characterized by pH of 6.5–7.0, AL-K_2_O 6550 mg kg^-1^ and AL-P_2_O_5_ 1402 mg kg^-1^. It is produced on natural clay carriers and naturally degradable granules of a water-retaining gel. 10 g pot^-1^ AMF inoculum was layered uniformly at a depth of 5 cm below the seeds in the mycorrhizal treatments.

The R_1_ microbial treatment was carried out with a peat-based soybean inoculum of *Bradyrhizobium japonicum* containing 10^6^ CFU g^-1^. A liquid culture of a *B. japonicum* strain from the strain collection of the Research Institute for Soil Sciences and Agricultural Chemistry was used for the R_2_ treatment. R_2_ *B. japonicum* strain was incubated on yeast extract mannitol agar (YMA) for 5 days (Vincent, 1970). After incubation, one loopful of the culture was suspended in 10 mL of sterile tap water. 100 mL of YMB (Yeast Extract Broth) culture medium was inoculated with this bacterial suspension and incubated for 5 days under continuous shaking at 28°C. In the case of rhizobial inoculation, the seedbed was inoculated with 1 mL suspension of R_1_ (2 g 200 mL^-1^ sterile tap water) or 1 mL liquid culture of R_2_. Each planting combination was represented by four replicates (*n* = 4).

### Drought Stress Experiment

The Aliz and Emese cultivars were tested for symbiotic effectiveness in chernozem soil treated with the previously selected microbial treatments. Besides the control, both cultivars were exposed to two treatments, co-inoculation with R_1_ *B. japonicum* chosen in the Compatibility experiment and F_1_ or F_2_ AMF inoculum. Plants growing with different water supplies were investigated in five-five replications (*n* = 5).

### Experimental Design and Growth Conditions

In the Compatibility experiment, the soybean seeds were planted in 72 1.25 dm^3^ plastic pots containing 1.25 kg of soil-analog ground pumice (porous vitroclastic perlite) medium with 0.7–1.1 mm particle size and pH_H2O_ 6.5. Two seeds were planted in each pot and thinned to one after emergence. The pumice, which lacks indigenous rhizobia and infective propagules of AM fungi, was treated with AMF or rhizobial inoculums, alone or in combination, except for the controls. The plants were cultivated in a random arrangement in a growth chamber for 65 days with day/night temperature and photoperiod of 26/18°C and 16/8 h respectively, at a photon flux density of 600 μmol m^-2^ s^-1^ and relative humidity of 50–70%. Optimal plant nutritional status was maintained by weekly irrigation with 100 ml of modified Hoagland’s solution (0.5 M KH_2_PO_4_) per pot.

The Drought Stress experiment was designed to check the results of the Compatibility experiment in organically managed haplic chernozem soil (IUSS Working Group WRB, 2015) that contains an indigenous AMF community. Pre-germinated soybean seeds were transplanted into 3.5 dm^3^ plastic pots filled with 3.9 kg of soil in five replicates (*n* = 5). The experimental soil, collected in an organic farming system in Martonvásár, Hungary (N47°18′41″, E18°46′48″, 109 m asl.), has a clay loam texture (USDA, 32.8% sand, 42.2% silt, 25.0% clay) with pH_H2O_ 7.69, pH_KCl_ 7.14, ammonium lactate acetate (AL) extractable P_2_O_5_ 371 mg kg^-1^, AL-K_2_O 402 mg kg^-1^, NH_4_^+^-N 4.06 mg kg^-1^, NO_3_^-^-N 17.47 mg kg^-1^, humus 2.93%, and 0.332 and 0.156 cm^3^ cm^-3^ water content at field capacity and permanent wilting point, respectively.

The cultivars were grown for 65 days with the chosen highly effective combinations of microbial partners (F_1_R_1_; F_2_R_1_) under climatic and light conditions identical to those in the Selection experiment. Half of the plants were watered adequately (200 ml per pot three times a week) while the others were put through two drought cycles during 23–35 and 40–60 days after planting (DAP). At the beginning of the drought cycles, water was withheld till the soil moisture decreased near to wilting point (requiring 3–6 days, depending on plant size), after which this water status was maintained by daily irrigation during the rest of the drought cycle.

### Pre-harvest Investigations

The effects of non-lethal water deficit during the growth of soybean cultivars prior to the reproductive phase were assessed by recording the number of nodes (NN) on the main stem, measuring the stem height (SH) and the relative water content (RWC) of the leaves, and by examining DSS on the leaves (symptoms of wilting and leaf loss/shedding on a 0–3 scale). Sampling was performed every 7th day from the 14th day after planting (DAP) until the beginning of the reproductive growth stage (R_1_). Data representing the functional aspects of the AMF-rhizobia-soybean symbiotic systems were obtained *in situ* by measuring chlorophyll fluorescence induction and root electrical capacitance (C_R_).

#### Chlorophyll Fluorescence Induction

A FMM Chlorophyll-A fluorometer (Barócsi et al., 2009) was used to measure the fluorescence induction parameters *in situ*. The internal light source was a 635 nm laser diode (QL63H5SA, Roithner Lasertechnik GmbH, Wien, Austria) with 20 mW maximum optical power. Traditional Kautsky induction kinetic curves were detected simultaneously at 690 nm (red) and 735 nm (far-red) wavelengths, where Chl-A fluorescence shows two maxima in leaves. Minimal (F0) and maximal fluorescence (Fm) values were detected, after which the light-adapted, steady-state quantum efficiency of photosynthetic electron transport, F_v_/F_m_ = (F_m_–F_0_)/F_m,_ was calculated for both emission maxima. The F_v_/F_m_ values showed a similar tendency at both wavelengths, so only the results for 690 nm are shown. The F_v/_F_m_ data were measured on all the plants on 63 DAP.

#### Root Electrical Capacitance (C_R_) Measurement

The extension and activity of the root system (rhizosphere) was assessed simply *in situ* by C_R_ measurement (Chloupek, 1972; Cseresnyés et al., 2016). When an alternating current passes through the root tissue, charge accumulation, i.e., polarization, occurs. The amount of electric charge stored by the root system can be expressed as electrical capacitance (in nanofarads) which is proportional to the active root surface area. The method is only valid for the comparison of plants of the same species grown in the same substrate, at the same moisture level (Chloupek et al., 2010). C_R_ measurements were carried out on all the plants on DAP 64 (before harvest and after the second drought stress period in the Drought Stress experiment) using a GW-8101G LCR instrument (GW Instek Co., Ltd., Taiwan) at 1 kHz frequency with 1 V terminal voltage. One terminal of the instrument was connected to the plant stem with a spring tension clamp fixed 10 mm above the substrate level, while the second was grounded by a stainless steel rod (6 mm ID, 15 cm long) inserted into the substrate. Electrocardiograph paste (Vascotasin^®^; Spark Promotions Co., Ltd., Budapest, Hungary) was smeared around the stem to maintain electrical contact, and the substrate was irrigated to field capacity before measurement.

#### Leaf Relative Water Content (RWC)

In the Drought Stress experiment, the relative water content (RWC) of the leaves was measured during drought exposure on DAP 22 and DAP 63 (González and González-Vilar, 2007).

### Post-harvest Measurements

Both the inoculated and control soybeans were harvested and sampled for microbial investigations after 65 days of cultivation (R1, R2 flowering stage and R3 green pod formation) (Ohyama et al., 2013). LA was measured using image processing. In both experiments, SDW and RDW were determined after drying the samples at 80°C for 48 h. The susceptibility to symbiotic associations and the dependence of soybean cultivars on microbial treatments were characterized by the index of RMID. RMID is an extension of the relative field mycorrhizal dependence (RFMD) index (Plenchette et al., 1983), taking into consideration all symbionts rather than just AMF, and is defined as RMID = 100 ^∗^[(dry weight of inoculated plant)-(dry weight of non-inoculated plant)]/dry weight of inoculated plant.

#### Quantification of AMF Root Colonization, Nodulation and Functionality of Rhizobia

After harvest, the soybean response to rhizobial inoculation was characterized by a number representing the density of nodules found on the primary roots and lateral root zones.

The nitrogenase enzyme activity of the nodules was measured by means of ARA (Hardy et al., 1968). After removing them from the pots, the 65-day-old plants were carefully washed, then the whole, nodulated roots were placed in a 500 ml glass bottle capped with a rubber septum. After adding 50 cm^3^ of acetylene gas (C_2_H_2_, >99.95% purity, Lindegas) to the bottle, the roots were incubated for 30 min at room temperature (22^°^C). A gas sample (500 μl) was removed using a gas-tight syringe and analyzed on a gas chromatograph (GC 8000, FISONS Instruments) with the following specifications: flame ionization detector (FID) and Porapak T column to separate ethylene from acetylene, carrier gas N_2_ = 175 kPa, hydrogen at 50 kPa, air at 80 kPa, injector temperature 100°C, oven temperature 80°C, isotherm, detector temperature 150°C. The area under the peaks was evaluated with Chrom-Card software and a standard of 10 ppm ethylene (C_2_H_4_) in N_2_ (Scotty 14, Supelco) was used for calibration.

After ARA, the roots were randomly sampled for AMF root colonization measurement performed after staining the separated root (≤1 mm indiameter) sub-samples with lactic acid-aniline blue according to Phillips and Hayman (1970). AMF colonization was estimated using a BX51 microscope (40-200X; Olympus Corp., Tokyo, Japan). The frequency (F%), the intensity of colonization (M %) and the arbusculum richness (A%) in the roots were calculated using a five-class system (Trouvelot et al., 1986) after observing 30 fine root segments, each 1 cm in length.

#### Concentration of Nitrogen (N), Phosphorus (P) and Potassium (K) in Leaves

The N (%), P and K (mg kg^-1^) concentrations of the plants were measured after harvest. The P and K macroelement concentrations of the plants were assessed after wet digestion of air-dried plant samples with cc. HNO_3_ + cc. H_2_O_2_. The nitrogen content in the leaves was determined by the Kjeldahl method (Kjeldahl, 1883) after digesting the samples in sulfuric acid (cc. H_2_SO_4_). The leaf element contents were measured with an ICP-AES instrument (Jobin-Yvon, ULTIMA2).

### Statistical Analysis

Results were analyzed using two-way ANOVA or Kruskal–Wallis non-parametric test if the prerequisites of ANOVA did not fulfill. Bartlett test of homogeneity of variance and Shapiro–Wilk normality test with model residuals were carried out before ANOVA. *Post hoc* tests were also carried out: LSD (least significant differences) values were calculated after ANOVA analysis, while a pairwise *t*-test with the Holm method was applied after the Kruskal–Wallis test. The comparisons between means were performed at the significance level of *p* < 0.05. The relationship between C_R_ and RDW or LA were evaluated using simple regression analysis (*p* < 0.05 for each treatment).

## Results

### Selection Experiment

The negative osmotic potential significantly decreased seed germination (Table 1). The PEG solution retarded germ development, to the greatest extent for the cultivar Splendor. The GSI_6_ values ranged from 0.0% (Splendor) to 60.3% (Aliz). Cultivars Aliz and Emese had the highest GSI_6_ values, so these were assumed to have high drought stress tolerance in the early stages of plant development. There was no relationship between the 1000-grain weight or maturity group of the cultivars and the GSI levels.

### Compatibility Experiment

**FIGURE 1:**
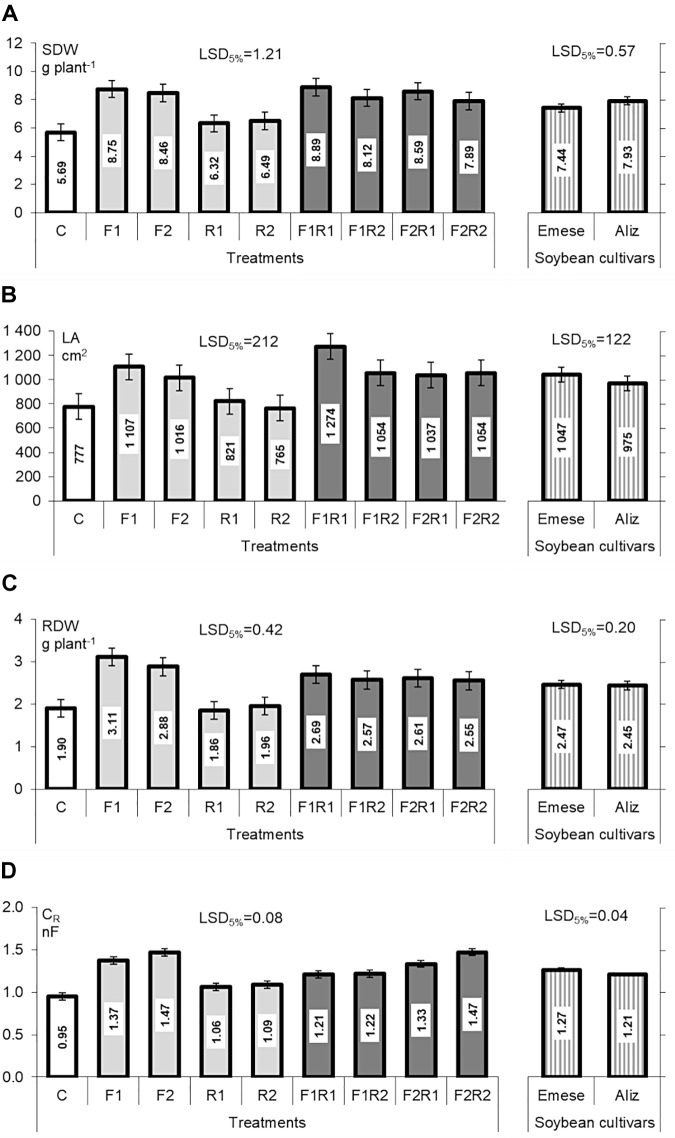
**(A)** Shoot dry weight (SDW), **(B)** leaf area (LA), **(C)** root dry weight (RDW), and **(D)** root electrical capacitance (C_R_) of Emese and Aliz soybean cultivars in the Compatibility experiment. Mean values of the microbial inoculation treatments (left side in the Figure: C, control plants; R_1_ and R_2_ rhizobia; F_1_ and F_2_ AM fungal products) and mean values of soybean cultivars (right side in the Figure). Error bars represent LSD (*n* = 4; *p* < 0.05).

In the Compatibility experiment all the AMF and dual inoculations resulted in higher SDW and RDW (Figures 1A,C), stem length and number of nodes (data not shown), especially in the F_1_ AMF treatments. The biomass production of AM plants was significantly higher (by 50–70%) than that of the controls or plants inoculated only with rhizobium. No significant differences in SDW and RDW were found between the soybean cultivars, though the SDW of Aliz was slightly higher than that of Emese. Aliz grew taller and were spindlier. The LA of the differentially inoculated soybean plants showed a tendency similar to their shoot biomass and dry weights (Figure 1B), except that Emese had higher LA than Aliz. No AMF structures were detected in the control or rhizobium inoculated plants (C and R_1,2_). Microscopic examination of the harvested roots showed a low frequency (F%) of fungal colonization in mycorrhizal plants irrespective of the AMF products.

A comparison of F_1_ and F_2_ showed that the former caused a greater increase in host growth, while the latter resulted in a higher colonization rate [F_1_max(F% = 1.67); F_2_max(F% = 19.84)]. AMF root colonization decreased in plants co-inoculated with both rhizobial and AMF products. No significant differences in AMF infectivity were found for the different host plant cultivars.

*Bradyrhizobium* inoculation resulted in intensive nodule formation on both the main root and lateral roots in all cases (100–150 nodules plant^-1^), but there were no significant differences between either the rhizobial treatments or the soybean cultivars. The ARA revealed that R_1_ had 2–5 times higher nitrogen-fixing capability (R_1_: 685 nm C_2_H_4_ h^-1^ pot^-1^; F_1_R_1_: 633 nm C_2_H_4_ h^-1^ pot^-1^; F_2_R_1_: 623 nm C_2_H_4_ h^-1^ pot^-1^) than R_2_ (R_2_: 252 nm C_2_H_4_ h^-1^ pot^-1^; F_1_R_2_: 144 nm C_2_H_4_ h^-1^ pot^-1^; F_2_R_2_: 199 nm C_2_H_4_ h^-1^ pot^-1^; LSD = 198, *P* < 0.05). There were no significant differences in the specific activity of the nitrogenase in the rhizobium-inoculated cultivars. The RMID of rhizobium- inoculated soybeans (R_1,2_) was lower than that of AMF- or co-inoculated plants, and Emese showed higher dependence than Aliz (except in F_2_R_2_) in well-watered pumice (data not shown).

The N content of plants treated only with rhizobium (R_1_; R_2_ in the case of Aliz) was significantly higher than that of control (C) and AMF-inoculated (F_1;_ F_2;_ F_1_R_1_; F_1_R_2_; F_2_R_1_; F_2_R_2_) hosts (Table 2). The leaf N content of Aliz was significantly higher, than that of Emese in the F_1_, R_2_, F_1_R_1_ and F_2_R_2_ treatments. The P concentration was generally higher in the leaves of Emese, except for the F_1_ treatment where a significant increment occurred in Aliz (Table 3).

**Table 2:** Nitrogen (N) content (%) of soybean leaves at the end of the Compatibility experiment.

N (%)	Cultivar: Emese	Cultivar: Aliz	LSD_5%_ 0.30
C	2.48 ± 0.44	2.50 ± 0.34	2.49
F_1_	2.24 ± 0.51	2.69 ± 0.29	2.47
F_2_	2.27 ± 0.21	2.22 ± 0.25	2.25
R_1_	2.96 ± 0.19	2.85 ± 0.12	2.91
R_2_	2.45 ± 0.03	3.09 ± 0.11	2.77
F_1_R_1_	2.47 ± 0.15	2.94 ± 0.35	2.71
F_1_R_2_	2.69 ± 0.11	2.79 ± 0.39	2.74
F_2_R_1_	2.70 ± 0.39	2.51 ± 0.43	2.61
F_2_R_2_	2.47 ± 0.29	2.73 ± 0.24	2.60

**LSD_5%_ 0.14**	2.53	2.70	**Mean values**



*Means and standard errors of four replicates. Two-way analysis of variance was performed. LSD_*5%*_ values indicate the least significant difference at the *p* = 0.05 level. Microbial treatments: C, non-inoculated control plants; F_*1*,2_, commercial AMF inocula; R_*1*_,_*2*,_*Bradyrhizobium japonicum* inocula; Soybean cultivars: Aliz and Emese.*

**Table 3:** Phosphorus (P) concentration (mg kg^-1^) of soybean leaves at the end of the Compatibility experiment.

P ( mg kg^-1^)	Cultivar: Emese	Cultivar: Aliz	LSD_5%_ 298
C	1629 ± 126	1593 ± 245	1611
F_1_	1889 ± 307	1907 ± 361	1898
F_2_	1599 ± 326	1325 ± 343	1462
R_1_	1825 ± 138	1466 ± 137	1645
R_2_	1465 ± 137	1512 ± 188	1488
F_1_R_1_	1649 ± 320	1634 ± 227	1641
F_1_R_2_	1703 ± 206	1644 ± 513	1674
F_2_R_1_	1522 ± 272	1259 ± 343	1390
F_2_R_2_	1365 ± 373	1396 ± 443	1381

**LSD_5%_ 141**	1627	1526	**Mean values**



*Means and standard errors of four replicates. Two-way analysis of variance was performed. LSD_*5%*_ values indicate the least significant difference at the *p* = 0.05 level. Microbial treatments: C, non-inoculated control plants; F_*1,2*_, commercial AMF inocula; R_*1*_,_*2*_, *Bradyrhizobium japonicum* inocula; Soybean cultivars: Aliz and Emese.*

Root electrical capacitance (C_R_) was the highest in AMF-treated plants (Figure 1D) for both Emese and Aliz. Significant differences in C_R_ were found between the mycorrhizal and non-mycorrhizal (C, R_1_, R_2_) plants. Rhizobium treatment caused a lower, but still significant increase in C_R_.

The photochemical efficiency (F_v_/F_m_) differed only slightly among the microbial treatments. R_1,_ F_1_R_1_ and F_1_R_2_ inoculation resulted in significantly higher F_v_/F_m_ ratios than the control (C: 0.827; R_1_: 0.839; F_1_R_1_,_2_: 0.840, 0.843 respectively; LSD_5%_ = 0.011). Photochemical efficiency also differed between the two varieties (Aliz: 0.836; Emese 0.827; LSD_5%_ = 0.006).

### Drought Stress Experiment

**FIGURE 2:**
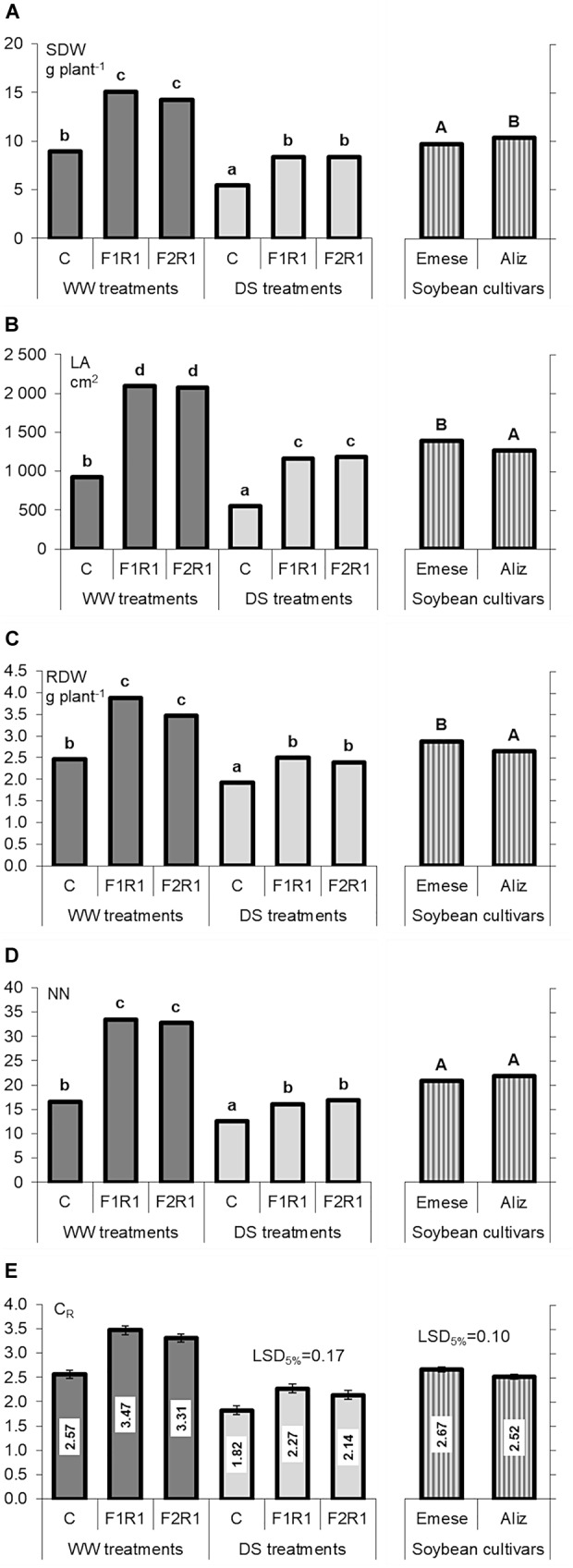
Changes in **(A)** shoot dry weight (SDW), **(B)** leaf area (LA), **(C)** root dry weight (RDW), **(D)** node number (NN), and **(E)** root:shoot ratio (RSR) of two soybean cultivars (Emese and Aliz) exposed to well-watered (WW) and drought-stressed (DS) conditions. Error bars represent LSD values, different letters mean significant differences (in case of transformed data) at *p* < 0.05 (*n* = 5).

Microbial inoculation caused a significant increment in SDW, LA, RDW (Figures 2A–C) and NN (except in F_1_R_1_) (Figure 2D) under both well-watered (WW) and drought stress (DS) conditions. The beneficial efficiency of the F_1_ was mostly higher than that of F_2_ (Figure 2). The microsymbionts induced a more than two-fold increase in LA (WW: 126–128%; DS: 113–115%) (Figure 2B). The LA of Emese was generally higher than that of Aliz. RDW was enhanced by 41–58% (WW) and 24–30% (DS) by co-inoculation (Figure 2C). Greater changes were observed in shoot than in root biomass production: inoculation increased SDW by 60–69% (WW) and 54% (DS).

The RSR values of microbially treated and control plants were statistically similar under WW conditions (Figure 2E).

Shoot dry weight decreased by 41% in the control and by 41–45% in the co-inoculated plants (Figure 2A). LA was 40% smaller in the control and 44% smaller in the co-inoculated plants (Figure 2B), while RDW was reduced by 22% in the control and by 31–36% in the co-inoculated plants (Figure 2C). NN also decreased significantly under drought stress (Figure 2D). SH was 21–24% lower after inoculation in the case of DS.

Combined inoculation with AM fungi and rhizobia was significantly more beneficial for Emese than for Aliz, as shown by differences in various plant growth parameters (LA: 10%, RSR: 15%) (Figures 2B,E). By contrast, the SDW of Aliz was higher than that of Emese. DS induced a greater decrease in SDW (35% Emese and 45% Aliz) than in RDW (18% Emese and 40% Aliz). No differences were found between the effects of F_1_ and F_2_ AMF inocula on plant growth. The drought-induced decline in LA ranged from 36% (Emese F_2_R) to 50% (Aliz F_2_R). Water deficit increased the RSR values in all the treatments and led to significant differences between the RSR values of inoculated and control plants, which were absent under WW conditions. Cultivar Emese exhibited significantly higher RSR than Aliz (Figure 2E). The growth parameters of F_1_ and F_2_ treated plants were statistically similar under DS.

**FIGURE 3:**
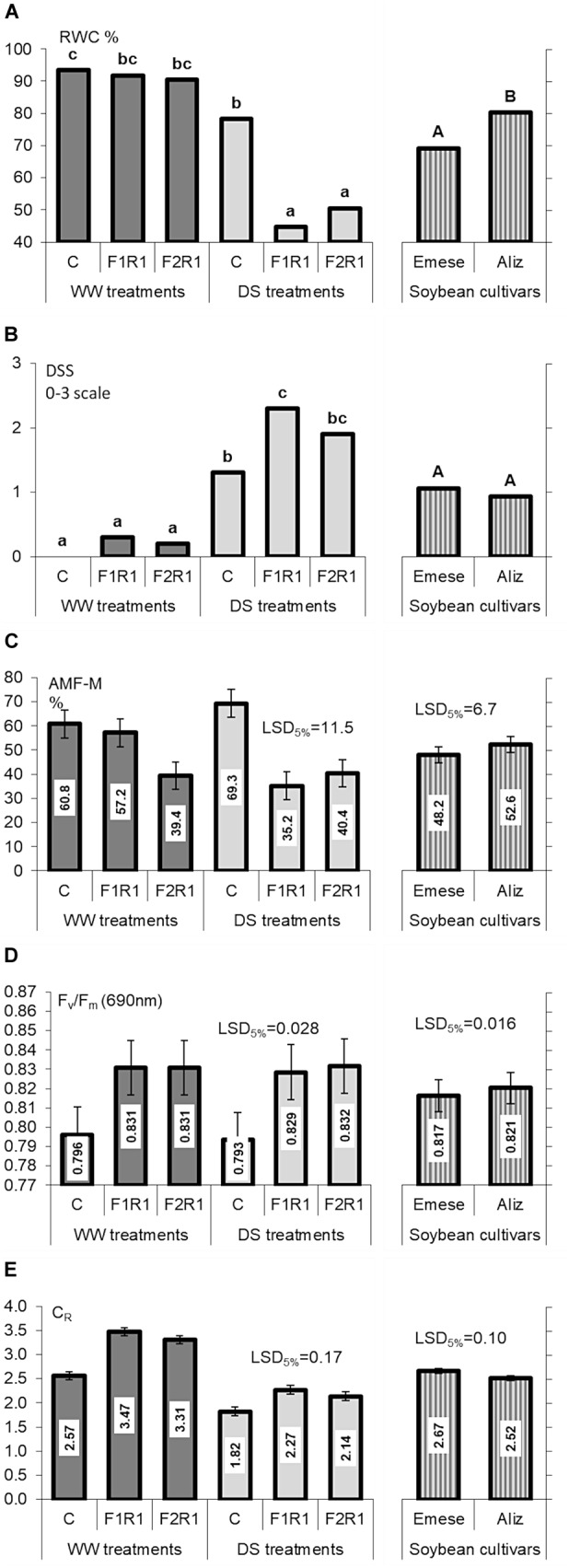
**(A)** Leaf relative water content (RWC), **(B)** drought-induced stress symptoms (DSS), **(C)** AMF colonization (M%), **(D)** chlorophyll-a fluorescence (F_v_/F_m_) and **(E)** root electrical capacitance (C_R_) of control (C) and co-inoculated (F_1_R_1_, F_2_R_2_) soybeans for different cultivars (Emese, Aliz) under well-watered (WW) and drought-stressed (DS) conditions. Error bars represent LSD values, different letters mean significant differences (in case of pairwise *t*-test) at *p* < 0.05.

There were no significant differences in RWC between microbially treated and control plants under WW conditions, irrespective of the cultivar (Figure 3A). The exposure of plants to drought led to a noticeable decrease in leaf RWC from 90.4–93.4% to 44.7–78.2% with significantly lower values for inoculated cultivars than for non-inoculated ones. Cultivar Aliz generally had higher RWC values in the vegetative phenophase, especially when inoculated before DS.

No differences were found in the DSS of the two cultivars in any treatment, though plants inoculated with F_1_ exhibited pronounced wilting symptoms (Figure 3B).

Fluorescein diacetate (FDA) hydrolysis revealed that the activity of soil microbes was significantly inhibited by DS in all the treatments. No significant effect of co-inoculation was observed for either WW (28.9–30.8 μg Fl g^-1^h^-1^; LSD = 4.2, *P* < 0.05) or DS (20.0–22.8 μg Fl g^-1^h^-1^). The FDA activity was also independent of the cultivar (Emese 24.2 μg Fl g^-1^h^-1^; Aliz 26.5 μg Fl g^-1^h^-1^; LSD = 2.4, *P* < 0.05) in both watering regimes.

The number of nodules on the roots of co-inoculated plants was much higher than on non-inoculated, control plants. Nodulation was independent of both the application of mycorrhizal fungi and the cultivar under both watering regimes.

The intensity of colonization (M%) in the roots ranged from 35.2 % to 60.8% in WW soil, with the lowest rate for F_2_ (Figure 3C). The M% of roots colonized by indigenous AMF exhibited a 9% increase in response to DS, while those colonized by AMF species from commercial products had suppressed or unchanged infectivity. Indigenous AMF alone generated a higher colonization in roots than it was observed after the inoculation with F_1_ or F_2_. The intensity of AMF colonization in the roots was slightly higher for Aliz than for Emese. The characteristics of and changes in arbusculum richness (A%) were similar to those of M%, again proving the better natural infectivity and responsiveness of Aliz. The increment in A% caused by DS was significant (data not shown). The RMID values for mycorrhizal and rhizobial treatments of the cultivars were greater under WW than under DS conditions. Under WW conditions, Emese had slightly higher susceptibility to microbial inoculation than Aliz, but DS inverted this relationship.

F_v_/F_m_ values were increased by dual inoculation under both water regimes (Figure 3D). Drought did not significantly affect the photosynthetic activity, and the quantum efficiency of PSII was independent of the cultivar.

**FIGURE 4:**
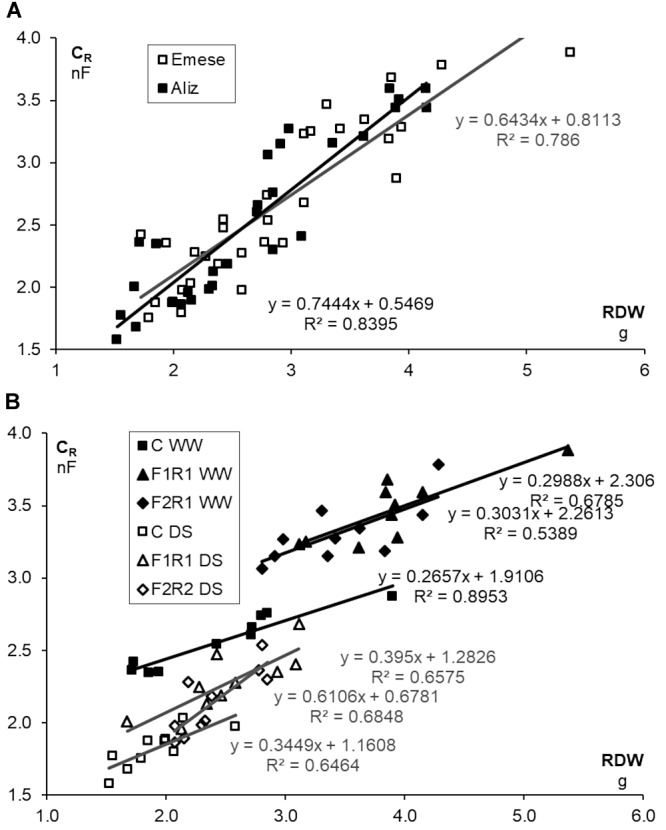
**(A)** Relationship between root electrical capacitance (C_R_) and root dry weight (RDW) of soybean cultivars (Emese, Aliz) and **(B)** RDW of control and co-inoculated (F_1_R_1_, F_2_R_1_) soybeans for different cultivars (Emese, Aliz) under well-watered (WW) and drought-stressed (DS) conditions.

Microbial inoculation significantly enhanced the C_R_ values of the cultivars, irrespectively of watering, particularly in the case of Emese (Figure 3E). The effect of F_1_ on C_R_ (36% for Aliz, 34% for Emese under WW; 21% for Aliz and 28% at Emese under DS) was greater than that of F_2_ (26% for Aliz, 32% for Emese under WW; 14% for Aliz and 20% for Emese under DS) (Figure 3D). C_R_ decreased significantly in response to DS, to a greater extent for Aliz (31–37%, as a percentage of WW) than for Emese (27–33%). Cultivar Emese exhibited significantly higher C_R_ than Aliz at the end of the experiment. A good linear correlation (*P* < 0.01) was obtained between C_R_ and both SDW (*R*^2^ = 0.829) and LA (*R*^2^ = 0.716) by pooling the data of the six plant groups. Strong linear correlations were found between both C_R_ and RDW and C_R_ and LA (Aliz *R*^2^ = 0.672; Emese *R*^2^ = 0.758). Figures 4A,B illustrate an increase in C_R_ due to inoculation and a decrease due to drought stress. Regression analysis demonstrated linear correlations between C_R_ and RDW for each treatment. The same root biomass was associated with higher C_R_ in inoculated treatments and with lower values in the case of drought stress (Figure 4B).

The beneficial effect of rhizobial nodulation on the leaf nitrogen concentration was detected, as in the Selection experiment. The leaf N percentage of inoculated plants significantly exceeded the controls under both WW and DS conditions (Table 4), but no significant differences were found either between the cultivars or between the microbial treatments. The leaf P concentration was decreased by drought and by inoculation under WW conditions, although DS led to higher leaf P concentration in inoculated plants compared to controls (Table 5). The total P content in the leaves was significantly higher in inoculated plants than in control plants. The P concentrations in Emese were greater than in Aliz.

**Table 4:** Nitrogen (N) content (%) of soybean leaves at the end of the Drought stress experiment.

N (%)		Cultivar: Emese	Cultivar: Aliz	LSD_5%_ 0.36
WW	C	1.33 ± 0.33	1.51 ± 0.81	1.42
	F_1_R_1_	3.12 ± 0.27	2.96 ± 0.24	3.04
	F_2_R_1_	3.08 ± 0.26	3.27 ± 0.32	3.18
DS	C	1.49 ± 0.39	1.20 ± 0.19	1.34
	F_1_R_1_	3.04 ± 0.23	2.93 ± 0.47	2.99
	F_2_R_1_	3.06 ± 0.26	3.10 ± 0.54	3.08

**LSD_5%_ 0.21**		2.52	2.49	**Mean values**



*Means and standard errors of five replicates. Two way-analysis of variance was performed. LSD_*5%*_ values indicate the least significant difference at the *p* = 0.05 level. Microbial treatments: C, non-inoculated control plants; F_*1,2*_, commercial AMF inocula; R_*1*_,_*2*_, *Bradyrhizobium japonicum* inocula; Soybean cultivars: Aliz and Emese; WW, well-watered conditions; DS, drought stress conditions.*

**Table 5:** Phosphorus (P) concentration (mg kg^-1^) of soybean leaves at the end of Drought stress experiment.

P (mg kg^-1^)		Cultivar: Emese	Cultivar: Aliz	LSD_5%_ 333
WW	C	3871 ± 780	3155 ± 345	3513
	F_1_R_1_	2693 ± 350	2300 ± 126	2496
	F_2_R_1_	2523 ± 131	2360 ± 118	2441
DS	C	3091 ± 334	3342 ± 616	3217
	F_1_R_1_	2744 ± 344	2714 ± 271	2729
	F_2_R_1_	2968 ± 103	2682 ± 226	2825

**LSD_5%_ 192**		2981	2759	**Mean values**



*Means and standard errors of five replicates. Two-way analysis of variance was performed. LSD_*5%*_ values indicate the least significant difference at the *p* = 0.05 level. Microbial treatments: C, non-inoculated control plants; F_*1,2*_, commercial AMF inocula; R_*1*_,_*2*_, *Bradyrhizobium japonicum* inocula; Soybean cultivars: Aliz and Emese; WW, well-watered conditions; DS, drought stress.*

## Discussion

For the future of mycorrhizal biotechnology and industry, it is crucial to incorporate the scientific knowledge derived from fundamental and applied research into the innovation of microbial inoculants (Vosátka et al., 2012). Although various commercial microbial products are already in use, few data have been published on their effect on plant development, nutrition and yield under controlled environmental conditions (Bona et al., 2016). Most studies have been primarily focused on the investigation of qualitative and quantitative yield indicators in horticulture, fruit or ornamental production (Albrechtova et al., 2012; Bona et al., 2016; Engel et al., 2016).

In the present study symbiotic effectiveness was investigated with 20–20 measured parameters on drought-selected soybean hosts during their vegetative and early reproductive stages, with special regard to the compatibility of dual and tripartite symbiotic agents. Most of the measured parameters confirmed the beneficial effect of inoculation with symbionts on plant development and drought tolerance (Figures 1–4). Similar to other observations (Okereke et al., 2001; Meghvansi et al., 2008), the responses of soybean to microbial inoculation depended considerably on the rhizobial strains, on the fungal products and also on the cultivars. The present investigations showed that the benefits of symbiosis were more obvious in the case of plants singly or co-inoculated with AMF than for those inoculated solely with rhizobium (Figure 1 and Tables 1, 2). After single inoculation with products containing AM fungal species (F_1_ and F_2_) the increase in shoot and RDW, LA and C_R_ was higher than in rhizobium only treatments (Figures 1A–D). With the exception of C_R_ these parameters differed slightly between soybean varieties. Such functional differences also occurred between the AMF treatments as reported earlier (Louis and Lim, 1988; Antunes et al., 2006; de Varennes and Goss, 2007). Infection with *Bradyrhizobium* clearly enhanced the leaf nitrogen content, but AMF colonization reduced the concentration of nitrogen and phosphorus due to the dilution effect of higher plant biomass (Tables 1, 2). In the Compatibility experiment, the results obtained for ARA and plant nitrogen content revealed clear differences in the effect of the two rhizobial inoculants on plant nutrition. Despite their lower colonization values, plants treated with the AMF inoculum F_1_ had a higher growth rate than those inoculated with F_2_ (Figures 1A–C). No AM fungal colonization was observed during the experimental period on the great majority of the roots in F_1_-treated pots. The extra biomass production caused by F_1_ could be due to the high organic content of the biofertilizer. The higher number of species in the F_2_ product could lead to higher infectivity and effectiveness, so F_2_ could be more successful in developing a compatible relationship.

Numerous field and pot trials proved that AMF inoculation significantly increased soybean yields (by 20–50%); however, the response varied with soil type, fertilization and the origin of the symbiotic partners (Porcel and Ruiz-Lozano, 2004; Meghvansi et al., 2008; Kaschuk et al., 2010; Wang et al., 2011). It was found that inoculation with *Bradyrhizobium* sp. or *Glomus mosseae* in pot culture was able to inhibit both pathogenic infection with *Cylindrocladium parasiticum* and the development of the parasite in soybean roots, while co-inoculation was more effective than the use of any of the microsymbionts alone (Gao et al., 2012). The positive interaction between the microsymbionts was indicated by the extra biomass production, the increased number of nodules and the richness of AMF root structures (Louis and Lim, 1988; Xie et al., 1995). However, the carbon cost of mycorrhizal fungi requires the delivery of 4–20% of the photosynthetically fixed carbon from the host plants to the symbiotic partner (Grimoldi et al., 2006; Kaschuk et al., 2009). According to Minchin et al. (1981), as much as 25% of net photosynthates may be appropriated for biological nitrogen fixation. Furthermore, the cost of two independent microsymbiotionts in tripartite associations may be cumulative (Harris et al., 1985). Although multiple inoculation may result in decreased biomass production or unfavorable microbial parameters, the present results indicated that microbial inoculation can improve photosynthetic efficiency both in well-watered soils and under drought stress, though this effect depends on the inoculants (Figure 3D). The increased photosynthetic activity and root functionality of bacteria or AMF treated hosts resulted in higher biomass production compared to control plants. However, co-inoculation with rhizobial and AM fungal strains did not produce significantly further more biomass in pumice (Figure 1A); moreover, in the case of dual inoculation, AMF root colonization exhibited a slight decrease.

Fluorescence kinetics and the root electrical capacitance methods proved to be useful tools for the *in situ* monitoring of the effect of several stress factors and for the selection of stress-tolerant cultivars or effective microbial strains (Solti et al., 2014; Cseresnyés et al., 2016, 2018), but less research has targeted the characterization of differences between different genotypes of crop species (Barócsi, 2013). The rate of photosynthesis is influenced by environmental conditions (e.g., water, temperature, nutrients, light and CO_2_) and internal factors, such as the nutrient concentration of the tissues and the sink strength stimulated by the carbon cost of symbiotic associations (Kaschuk et al., 2009). In the present experiments significant differences were found both in the chlorophyll fluorescence and C_R_ values of different microbial treatments and soybean cultivars. Of the two varieties, the Aliz had higher photochemical efficiency, which represented an inverse tendency compared to the LA values. Both in pumice and organic soil C_R_ was closely correlated with root biomass (Cseresnyés et al., 2016). However, the measured values were the joint result of root activity, the activity of root-associated symbionts and the soil. This means that at the same stage of development, under similar soil conditions, both rhizobial nodules and AMF extraradical hyphal network may have a beneficial effect on active root surface area. The same root biomass is associated with higher C_R_ in the case of inoculated plants and with lower values in response to drought stress (Cseresnyés et al., 2013). The increased water uptake of inoculated plants is presumably due to the enhanced root–soil interface caused by the external fungal hyphae and the root nodulation. It was found earlier that eight soybean cultivars could be classified into different drought-tolerance groups on the basis of C_R_ showing a strong correlation with cultivar-specific root growth and biomass production under both well-watered and drought conditions (Cseresnyés et al., 2016). Several studies proved that advanced root properties such as greater depth and larger root system with more root hairs are advantageous under water deficit (Ku et al., 2013; Kumagai and Sameshima, 2014; Cseresnyés et al., 2016). It was also found that the effects of co-inoculation were related to the root morphology of soybean genotypes (Wang et al., 2011, 2016). For example, deeply rooted hosts benefited more than shallowly rooted ones. DS impeded plant growth in all the treatments for both cultivars (Figures 2A–D) (Porcel and Ruiz-Lozano, 2004; Masuda and Goldsmith, 2009). Similar to other investigations (Ruiz-Lozano et al., 2001; Porcel and Ruiz-Lozano, 2004), mycorrhizal associations seem to have played an important role in the drought stress tolerance of soybean. Strong linear C_R_-RDW and C_R_-LA relationships were revealed in the Drought Stress experiment, (Figures 4A,B). As the leaves vaporize the water taken up by the roots, LA should be proportional to the absorptive surface area of the root system involved in the mycorrhizosphere, being manifested as a correlation between C_R_ and LA. In the case of drought stress, the beneficial effect of inoculation was mostly more pronounced in Emese than in Aliz. Emese produced a bigger root system and was more resistant to drought stress than Aliz, where the levels of RSR and C_R_ were lower (Figure 2E). Although Emese lost more water from its leaves due to the larger biomass, it did not differ from Aliz in terms of DSS (Figure 3B). The loss of biomass caused by drought was also lower in the case of Emese, while the mycorrhizal dependence of Aliz increased under drought. These results suggest that the functional properties of the roots may significantly affect the susceptibility of cultivars to microsymbionts, which could also influence the development of cultivar-specific stress tolerance (Wang et al., 2016). It has been reported that grasses having hairy and bushy fibrous roots with a large surface for uptake are less mycotroph than the tap-rooted plants in mycotrophic plant families (Muleta, 2010). In a tripartite symbiotic association, AM fungi respond more sensitively to differences in the (functional) properties of the root between species and varieties than rhizobia (Wang et al., 2016). The establishment of symbiosis enlarges the active root surface area responsible for water and nutrient uptake, which can be detected by measuring C_R_. C_R_ measurements are sufficiently sensitive to detect differences in the infectivity of AM strains (Takács et al., 2014). No data on the C_R_ of nodulated roots have yet been published. In the present experiments plants infected with rhizobium had significantly higher C_R_ than the control despite the similar RDW (Figure 4B). Nodules and the extraradical hyphal network add to C_R_ by enlarging the absorbing surface.

It should be noted that when evaluating the beneficial effects of inoculation one should allow for the carriers of the commercial products, which may contain nutrients. The extra biomass production caused by the F_1_ AMF biofertilizer could be the result of its high organic material content. Multifactorial investigations are needed for the determinination of host compatibility and the efficacy of microbial inocula on plant production, including the measurement of plant growth and functional parameters, with both *in situ* and destructive methods.

In the field, the symbiotic effectiveness of rhizobial strains or AMF inoculants and their competitiveness can be achieved by superior microsymbionts originated from natural or managed selection. However, no single AMF or rhizobium strain is likely to be effective in all soils under any conditions and on any plant host, therefore no commercial fertilizer can be expected to be ideal for every field, despite containing multiple strains or species. All the products need to be tested before application and it is possible that no suitable commercial fertilizer will be adequate for a specific purpose. In this case, the managed selection of strains originating from the indigenous microbial community could be the best solution.

In low-input agricultural systems, research on cooperation between different microbial symbionts is a key to understanding how the health and productivity of the plant is supported (Mahdi et al., 2010; Bhardwaj et al., 2014). Increasing the fitness and vitality of host plants to environmental stress by means of site-adapted, compatible symbiotic partnerships could be a new strategy for mitigating the impacts of environmental stress factors on plant production (Gianinazzi et al., 2010). Little differences in the root properties of drought tolerant cultivars may cause significant differences in the growth and physiological parameters that are used to describe symbiotic relationships. Endurance of even drought tolerant cultivars can be improved by inoculation with AMF or nitrogen fixing bacteria. In well-watered conditions, the tripartite association did not show the synergistic effect on the plants, so the benefits produced by commercial AMF products may have been partially originated from their carrier. The efficiency of these biofertilizers should therefore be checked before large scale application. The present results show the potential of C_R_ measurements to monitor the effect of symbiotic factors influencing root growth and biomass production. This *in situ* technique provides an opportunity to follow the temporal changes in root activity and to select efficient plant-microbe partnerships.

## Author Contributions

TT supervised the project, conducted the pot experiments, discussed the results, and wrote the paper. IC was responsible for C_R_ measurements and data evaluation. TT, RK, IP, BK, TS-K, and AF designed and carried out the investigations on the symbiotic microorganisms and on the plants. All authors read the manuscript and approved the submission.

## Conflict of Interest Statement

The authors declare that the research was conducted in the absence of any commercial or financial relationships that could be construed as a potential conflict of interest.

## Abbreviations

AMF: arbuscular mycorrhizal fungi
ARA: acetylene reduction assay
C_R_: root capacitance
DSS: drought stress symptoms
F_v_/F_m_: maximum quantum efficiency of photosystem II photochemistry
GSI: germination stress index
LA: leaf area
NN: node number
RDW: root dry weight
RMID: relative microbial inoculation dependence
RSR: root shoot ratio
RWC: leaf relative water content
SDW: shoot dry weight
SH: shoot height

## References

[B1] Albareda M. Rodríguez-Navarro D. N. Temprano F. J. (2009). Soybean inoculation: dose, N fertilizer supplementation and rhizobia persistence in soil. *Field Crops Res.* 113 352–356. 10.1016/j.fcr.2009.05.013

[B2] Albrechtova J. Latr A. Nedorost L. Pokluda R. Posta K. Vosatka M. (2012). Dual inoculation with mycorrhizal and saprotrophic fungi applicable in sustainable cultivation improves the yield and nutritive value of onion. *ScientificWorldJournal* 2012:374091. 10.1100/2012/374091 22666113 PMC3361196

[B3] Antunes P. M. Deaville D. Goss M. J. (2006). Effect of two AMF life strategies on the tripartite symbiosis with *Bradyrhizobium japonicum* and soybean. *Mycorrhiza* 16 167–173. 10.1007/s00572-005-0028-3 16362418

[B4] Appunuu C. Zoue A. N. Laguerre G. (2008). Genetic diversity of native Bradyrhizobia isolated from soybeans (Glycine max L.) in different agricultural-ecological-climatic regions of India. *Appl. Env. Ecol.* 74 5991–5996. 10.1128/AEM.01320-08 18676699 PMC2565974

[B5] Artusson V. Finlay R. D. Jansson J. K. (2006). Interactions between arbuscular mycorrhizal fungi and bacteria and their potential for stimulating plant growth. *Environ. Microbiol.* 8 1–10. 10.1111/j.1462-2920.2005.00942.x 16343316

[B6] Balog A. Loxdale H. D. Bálint J. Benedek K. Szabó K. A. Jánosi-Rancz K. T. (2017). The arbuscular mycorrhizal fungus *Rhizophagus irregularis* affects arthropod colonization on sweet pepper in both the field and greenhouse. *J. Pest Sci.* 90 935–946. 10.1007/s10340-017-0844-1

[B7] Barea J. M. (1997). “Mycorrhiza-bacteria interactions on plant growth promotion,” in *Plant Growth Promoting Rhizobacteria*, eds Ogoshi A. K. Kobayashi A. K. Homma Y. Kodama Y. F. Kondo N. Akino S. (Paris: OECD Press), 150–158.

[B8] Barócsi A. (2013). Intelligent, net or wireless enabled fluorosensors for high throughput monitoring of assorted crops. *Measure. Sci. Technol.* 24:025701 10.1088/0957-0233/24/2/025701

[B9] Barócsi A. Lenk S. Kocsányi L. Buschmann C. (2009). Excitation kinetics during induction of chlorophyll a fluorescence. *Photosynthesis* 47 104–111. 10.1007/s11099-009-0016-5

[B10] Behie S. W. Bidochka M. J. (2014). Nutrient transfer in plant-fungal symbioses. *Trends Plant Sci.* 19 734–740. 10.1016/j.tplants.2014.06.007 25022353

[B11] Bethlenfalvay G. J. Milford S. Brown K. L. M. Stafford A. E. (1987). *Glycine Glomus- Rhizobium* symbiosis. *Plant Physiol.* 85 115–119. 10.1104/pp.85.1.115 16665641 PMC1054214

[B12] Bhardwaj D. Ansari M. W. Sahoo R. K. Tuteja N. (2014). Biofertilizers function as key player in sustainable agriculture by improving soil fertility, plant tolerance and crop productivity. *Microb. Cell Fact.* 13:66. 10.1186/1475-2859-13-66 24885352 PMC4022417

[B13] Biró B. Köves-Péchy K. Vörös I. Takács T. Eggenberger P. Strasser R. J. (2000). Interrelations between *Azospirillum* and *Rhizobium* nitrogen fixers and arbuscular mycorrhizal fungi in the rhizosphere of alfalfa in sterile AMF free or normal conditions. *Appl. Soil Ecol.* 15 159–168. 10.1016/S0929-1393(00)00092-5

[B14] Bona E. Lingua G. Todeschini V. (2016). “Effect of bioinoculants on the quality of crops,” in *Bioformulations: For Sustainable Agriculture*, ed. Balestrini R. (New Delhi: Springer), 10.1007/978-81-322-2779-35

[B15] Bouslama M. Schapaugh W. T. (1984). Stress tolerance in soybeans. Part 1. Evaluation of three screening techniques for heat and drought tolerance. *Crop Sci.* 24 933–937. 10.2135/cropsci1984.0011183X002400050026x

[B16] Brundrett M. C. (2009). Mycorrhizal associations and other means of nutrition of vascular plants: understanding the global diversity of host plants by resolving conflicting information and developing reliable means of diagnosis. *Plant Soil* 320 37–77. 10.1007/s11104-008-9877-9

[B17] Cameron D. D. Neal A. L. van Wees S. C. Ton J. (2013). Mycorrhiza-induced resistance: more than the sum of its parts? *Trends Plant Sci.* 18 539–545. 10.1016/j.tplants.2013.06.004 23871659 PMC4194313

[B18] Cavagnaro T. R. Smith F. A. Smith S. E. Jakobsen I. (2005). Functional diversity in arbuscular mycorrhizas: exploitation of soil patches with different phosphate enrichment differs among fungal species. *Plant Cell Environ.* 28 642–650. 10.1111/j.1365-3040.2005.01310.x

[B19] Cely M. V. T. de Oliveira A. G. de Freitas V. F. de Luca M. B. Barazetti A. R. dos Santos I. M. O. (2016). Inoculant of arbuscular mycorrhizal fungi (*Rhizophagus clarus*) increase yield of soybean and cotton under field conditions. *Front. Microbiol.* 7:720. 10.3389/fmicb.2016.00720 27303367 PMC4880672

[B20] Chloupek O. (1972). The relationship between electric capacitance and some other parameters of plant roots. *Biol. Plant.* 14 227–230. 10.1007/BF02921255

[B21] Chloupek O. Dostál V. Støeda T. Psota V. Dvoøáèková O. (2010). Drought tolerance of barley varieties in relation to their root system size. *Plant Breed.* 129 630–636. 10.1111/j.1439-0523.2010.01801.x

[B22] Cseresnyés I. Rajkai K. Takács T. (2016). Indirect monitoring of root activity in soybean cultivars under contrasting moisture regimes by measuring electrical capacitance. *Acta Physiol. Plant.* 38:121 10.1007/s11738-016-2149-z

[B23] Cseresnyés I. Szitár K. Rajkai K. Füzy A. Mikó P. Kovács R. (2018). Application of electrical capacitance method for prediction of plant root mass and activity in field-grown crops. *Front. Plant Sci.* 9:93. 10.3389/fpls.2018.00093 29449861 PMC5799269

[B24] Cseresnyés I. Takács T. Végh K. R. Anton A. Rajkai K. (2013). Electrical impedance and capacitance method: a new approach for detection of functional aspects of arbuscular mycorrhizal colonization in maize. *Eur. J. Soil Biol.* 54 25–31. 10.1016/j.ejsobi.2012.11.001

[B25] de Varennes A. Goss M. J. (2007). The tripartite symbiosis between legumes, rhizobia and indigenous mycorrhizal fungi is more efficient in undisturbed soil. *Soil Biol. Biochem.* 39 2603–2607. 10.1016/j.soilbio.2007.05.007

[B26] Denison R. F. Kiers E. T. (2011). Life histories of symbiotic rhizobia and mycorrhizal fungi. *Curr. Biol.* 21 775–785. 10.1016/j.cub.2011.06.018 21959168

[B27] Domokos E. Jakab-Farkas L. Darkó B. Bíró-Janka B. Mara G. Albert C. (2018). Increase in artemisia annua plant biomass artemisinin content and guaiacol peroxidase activity using the arbuscular mycorrhizal fungus *Rhizophagus irregularis*. *Front. Plant Sci.* 9:478. 10.3389/fpls.2018.00478 29706981 PMC5908966

[B28] Engel R. Szabó K. Abrankó L. Rendes K. Füzy A. Takaìcs T. (2016). Effect of arbuscular mycorrhizal fungi on the growth and polyphenol profile of marjoram, lemon balm, and marigold. *J. Agric. Food Chem.* 64 3733–3742. 10.1021/acs.jafc.6b00408 27096876

[B29] Farooq M. Gogoi N. Barthakur S. Baroowa B. Bharadwaj N. Alghamdi S. S. (2016). Drought stress in grain legumes during reproduction and grain filling. *J. Agro. Crop Sci.* 203 81–102. 10.1111/jac.12169 21610017

[B30] Fauvart M. Michiels J. (2008). Rhizobial secreted proteins as determinants of host specificity in the rhizobium–legume symbiosis. *FEMS Microbiol. Lett.* 285 1–9. 10.1111/j.1574-6968.2008.01254.x 18616593

[B31] Gao X. Lu X. Wu M. Zhang H. Pan R. Tian J. (2012). Co-inoculation with rhizobia and AMF inhibited soybean red crown rot: from field study to plant defense-related gene expression analysis. *PLoS One* 7:e33977. 10.1371/journal.pone.0033977 22442737 PMC3307780

[B32] Gianinazzi S. Gollotte A. Binet M. N. van Tuinen D. Redecker D. Wipf D. (2010). Agroecology: the key role of arbuscular mycorrhizas in ecosystem services. *Mycorrhiza* 20 519–530. 10.1007/s00572-010-0333-3 20697748

[B33] González L. González-Vilar M. (2007). “Determination of relative water content,” in *Handbook of Plant Ecophysiology Techniques*, ed. Reigoza M. J. R. (Dordrecht: Kluwer Academic Publishers), 207–212. 10.1007/0-306-48057-3_14

[B34] Gosling P. Hodge A. Goodlass G. Bending G. D. (2006). Arbuscular mycorrhizal fungi and organic farming. *Agric. Ecosyst. Environ.* 113 17–35. 10.1016/j.agee.2005.09.009

[B35] Grimoldi A. A. Kavanová M. Lattanzi F. A. Schäufele R. Schnyder H. (2006). Arbuscular mycorrhizal colonization on carbon economy in perennial ryegrass: quantification by 13CO2/12CO2 steady-state labelling and gas exchange. *New Phytol.* 172 544–553. 10.1111/j.1469-8137.2006.01853.x 17083684

[B36] Hardy R. W. F. Holsten R. D. Jackson E. K. Burns R. C. (1968). Acetylene - ethylene assay for N2 fixation: laboratory and field evaluation. *Plant Physiol.* 43 1185–1207. 10.1104/pp.43.8.1185 16656902 PMC1086994

[B37] Harris D. Pacovsky R. S. Paul E. A. (1985). Carbon economy of soybean–rhizobium–glomus associations. *New Phytol.* 101 427–440. 10.1111/j.1469-8137.1985.tb02849.x 33874245

[B38] Herrera-Peraza R. A. Hamel C. Fernández F. Ferrer R. L. Furrazola E. (2011). Soil–strain compatibility: the key to effective use of arbuscular mycorrhizal inoculants? *Mycorrhiza* 21 183–193. 10.1007/s00572-010-0322-6 20552233 PMC3058370

[B39] Hodge A. Storer K. (2017). Arbuscular mycorrhiza and nitrogen: implications for individual plants through to ecosystems. *Plant Soil* 386 1–19. 10.1007/s11104-014-2162-1

[B40] IUSS Working Group WRB (2017). “World Reference Base (WRB) for Soil Resources 2014, update 2015,” in *Proceedings of the International soil classification system for naming soils and creating legends for soil maps. World Soil Resources Reports* 106 (Rome: FAO).

[B41] Jakobsen I. Abbott L. K. Robson A. D. (1992). External hyphae of vesicular-arbuscular mycorrhizal fungi associated with *Trifolium subterraneum* L. I. Spread of hyphae and phosphorus inflow into roots. *New Phytol.* 120 371–380. 10.1111/j.1469-8137.1992.tb01077.x

[B42] Johnson N. C. Graham J. H. Smith F. A. (1997). Functioning and mycorrhizal associations along mutualism-parasitism continuum. *New Phytol.* 135 575–586. 10.1046/j.1469-8137.1997.00729.x

[B43] Kaschuk C. Kuyper T. W. Leffelaar P. A. Hungria M. Giller K. E. (2009). Are the rates of photosynthesis stimulated by the carbon sink strength of rhizobial and arbuscular mycorrhizal symbioses? *Soil Biol. Biochem.* 41 1233–1244. 10.1016/j.soilbio.2009.03.005

[B44] Kaschuk G. Leffelaar P. A. Giller K. E. Alberton O. Hungria M. Kuyper T. W. (2010). Responses of legumes to rhizobia and arbuscular mycorrhizal fungi: a meta-analysis of potential photosynthate limitation of symbioses. *Soil Biol. Biochem.* 42 125–127. 10.1016/j.soilbio.2009.10.017

[B45] Kiers E. T. West S. A. Denison R. F. (2002). Mediating mutualisms: the influence of farm management practices on the evolutionary maintenance of symbiont co-operation. *J. Appl. Ecol.* 39 745–754. 10.1046/j.1365-2664.2002.00755.x

[B46] Kjeldahl J. Z. (1883). A new method for the determination of nitrogen in organic bodies. *Z. Anal. Chem.* 22 366–383. 10.1007/BF01338151

[B47] Ku Y.-S. Au-Yeung W.-K. Yung Y.-L. Li M.-W. Wen C.-Q. Liu X. (2013). “Drought stress and tolerance in soybean,” in *A Comprehensive Survey of International Soybean Research - Genetics, Physiology, Agronomy and Nitrogen relationships*, ed. Board J. E. (New York, NY: InTech), 209–237. 10.5772/52945

[B48] Kumagai E. Sameshima R. (2014). Genotypic differences in soybean yield responses to increasing temperature in a cool climate are related to maturity group. *Agric. Forest Meteorol.* 198–199, 265–272. 10.1016/j.agrformet.2014.08.016

[B49] Lisette J. Xavier C. Germida J. J. (2003). Selective interactions between arbuscular mycorrhizal fungi and *Rhizobium leguminosarum* bv. viceae enhance pea yield and nutrition. *Biol. Fertil. Soils* 37 261–267. 10.1007/s00374-003-0605-6

[B50] Louis I. Lim G. (1988). Differential response in growth and mycorrhizal colonisation of soybean to inoculation with two isolates of *Glomus clarum* in soils of different P availability. *Plant Soil* 112 37–43. 10.1007/BF02181750

[B51] Mahdi S. S. Hassan G. I. Samoon S. A. Rather H. A. Dar S. A. Zehra B. (2010). Bio-fertilizers in organic agriculture. *J. Phytol.* 2 42–54.

[B52] Marschner H. (1997). “The soil-root interface (rhizosphere) in relation to mineral nutrition,” in *Mineral Nutrition of Higher Plants*, ed. Marschner H. (London: Academic Press), 537–594.

[B53] Masuda T. Goldsmith P. D. (2009). World soybean production: Area harvested, yield, and long-term projections. *IAMA* 12 143–162.

[B54] Meghvansi M. K. Prasad K. Harwani D. Mahna S. K. (2008). Response of soybean cultivars toward inoculation with three arbuscular mycorrhizal fungi and *Bradyrhizobium japonicum* in the alluvial soil. *Eur. J. Soil Biol.* 44 316–323. 10.1016/j.ejsobi.2008.03.003

[B55] Menge D. N. L. Hedin L. O. Pacala S. W. (2012). Nitrogen and phosphorus limitation over long-term ecosystem development in terrestrial ecosystems. *PLoS One* 7:e42045. 10.1371/journal.pone.0042045 22870281 PMC3411694

[B56] Michel B. E. R. Kaufmann M. R. (1973). The osmotic potential of polyethylene glycol 6000. *Plant Physiol.* 51 914–916. 10.1104/pp.51.5.914 16658439 PMC366375

[B57] Miller A. J. Cramer M. D. (2004). Root nitrogen acquisition and assimilation. *Plant Soil* 274 1–36. 10.1007/1-4020-4099-7_1

[B58] Minchin F. R. Summerfield R. J. Hadley P. Roberts E. H. Rawsthorne S. (1981). Carbon and nitrogen nutrition of nodulated roots of grain legumes. *Plant Cell Environ.* 4 5–26. 10.1111/j.1365-3040.1981.tb00831.x

[B59] Muleta D. (2010). “Legume responses to arbuscular mycorrhizal fungi inoculation in sustainable agriculture,” in *Inoculation Microbes for Legume Improvement*, eds Khan M. S. Zaidi A. Musarrat J. (Wien: Springer), 293–323. 10.1007/978-3-319-59174-2_10

[B60] Munkvold L. Kjoller R. Vestberg M. Rosendahl S. Jakobsen I. (2004). High functional diversity within species of arbuscular mycorrhizal fungi. *New Phytol.* 164 357–364. 10.1111/j.1469-8137.2004.01169.x 33873553

[B61] Neves M. C. P. Rumjanek N. G. (1997). Diversity and adaptability of soybean and cowpea rhizobia in tropical soils. *Soil Biol. Biochem.* 29 889–895. 10.1016/S0038-0717(96)00205-2

[B62] OECD-FAO (2018). *OECD-FAO Agricultural Outlook 2018-2027.* Paris: OECD Publishing 10.1787/agr_outlook-2018-en

[B63] Ohyama T. Minagawa R. Ishikawa S. Yamamoto M. Hung N. V. P. Ohtake N. (2013). “Soybean seed production and nitrogen nutrition,” in *A Comprehensive Survey of International Soybean Research - Genetics, Physiology, Agronomy and Nitrogen Relationships*, ed. Board J. E. (Rijeka: InTech), 115–157. 10.5772/52287

[B64] Okereke G. U. Onochie C. Onunkwo A. Onyeagba E. (2001). Effectiveness of foreign bradyrhizobia strains in enhancing nodulation, dry matter and seed yield of soybean (*Glycine max* L.) cultivars in Nigeria. *Biol. Fertil. Soils* 33 3–9. 10.1007/s003740000264

[B65] Ossler J. N. Zielinski C. A. Heath K. D. (2017). Tripartite mutualism: Facilitation or trade-offs between rhizobial and mycorrhizal symbionts of legume hosts. *Am. J. Bot.* 102 1332–1341. 10.3732/ajb.1500007 26290556

[B66] Phillips J. M. Hayman D. S. (1970). Improved procedures for clearing roots and staining parasitic and vesicular-arbuscular mycorrhizal fungi for rapid assessment of infection. *Trans. Brit. Mycol. Soc.* 55 158–161. 10.1016/S0007-1536(70)80110-3

[B67] Plenchette C. Fortin J. A. Furlan V. (1983). Growth responses of several plant species to mycorrhizae in a soil of moderate P-fertility. *Plant Soil* 70 199–209. 10.1007/BF02374780

[B68] Porcel R. Ruiz-Lozano J. M. (2004). Arbuscular mycorrhizal influence on leaf water potential, solute accumulation, and oxidative stress in soybean plants subjected to drought stress. *J. Exp. Bot.* 55 1743–1750. 10.1093/jxb/erh188 15208335

[B69] Purcell L. C. King C. A. Ball R. A. (2000). Soybean cultivar differences in ureides and the relationship to drought tolerant nitrogen fixation and manganese nutrition. *Crop Sci.* 40 1062–1070. 10.2135/cropsci2000.4041062x

[B70] Rapparini F. Peñuelas J. (2014). “Mycorrhizal fungi to alleviate drought stress on plant growth,” in *Use of Microbes for the Alleviation of Soil Stresses*, ed. Miransari M. (New York, NY: Springer), 21–42. 10.1007/978-1-4614-9466-9_2

[B71] Ruiz-Lozano J. M. Collados C. Barea J. M. Azcón R. (2001). Arbuscular mycorrhizal symbiosis can alleviate drought-induced nodule senescence in soybean plants. *New Phytol.* 151 493–502. 10.1046/j.0028-646x.2001.00196.x

[B72] Schneider K. D. Derek H. Lynch K. Dunfield K. Khosla J. Jansa R. (2017). Farm system management affects community structure of arbuscular mycorrhizal fungi. *Appl. Soil. Ecol.* 96 192–200. 10.1016/j.apsoil.2015.07.015

[B73] Silvente S. Sobolev A. P. Lara M. (2012). Metabolite adjustments in drought tolerant and sensitive soybean genotypes in response to water stress. *PLoS One* 7:e38554. 10.1371/journal.pone.0038554 22685583 PMC3369847

[B74] Solti Á Lenk S. Mihailova G. Mayer P. Barócsi A. Georgieva K. (2014). Effects of habitat light conditions on the excitation quenching pathways in desiccating *Haberlea rhodopensis* leaves: an intelligent fluoro sensor study. *J. Photochem. Photobiol. B Biol.* 130 217–225. 10.1016/j.jphotobiol.2013.11.016 24345600

[B75] Takács T. Füzy A. Rajkai K. Cseresnyés I. (2014). Investigation of arbuscular mycorrhizal status and functionality by electrical impedance and capacitance measurement. *Acta Biol. Szeged.* 58 55–59.

[B76] Trouvelot A. Kought J. L. Gianinazzi-Pearson V. (1986). “Mesure du taux de mycorrhization VA d’un systéme radiculaire. Recherche de méthodes d’estimation ayant une signification fonctionnelle,” in *Ler Symposium Europeen sur les Mycorrhizes*, eds Gianinazzi-Pearson V. Gianinazzi S. (Paris: INRA), 217–221.

[B77] Vincent J. M. (1970). *A Manual for the Practical Study of the Root Nodule Bacteria.* Oxford-Edinburgh: Blackwell Scientific Publ.

[B78] Vosátka M. Látr A. Gianinazzi S. Albrechtová J. (2012). Development of arbuscular mycorrhizal biotechnology and industry: current achievements and bottlenecks. *Symbiosis* 58 29–37. 10.1007/s13199-012-0208-9

[B79] Wahbi S. Maghraoui T. Hafidi M. Sanguin H. Oufdou K. Prin Y. (2016). Enhanced transfer of biologically fixed N from faba bean to intercropped wheat through mycorrhizal symbiosis. *Appl. Soil Ecol.* 107 91–98. 10.1016/j.apsoil.2016.05.008

[B80] Wang X. Pan Q. Chen F. Yan X. Liao H. (2011). Effects of co-inoculation with arbuscular mycorrhizal fungi and rhizobia on soybean growth as related to root architecture and availability of N and P. *Mycorrhiza* 21 173–181. 10.1007/s00572-010-0319-1 20544230

[B81] Wang X. Zhao S. Bücking H. (2016). Arbuscular mycorrhizal growth responses are fungal specific but do not differ between soybean genotypes with different phosphate efficiency. *Ann. Bot.* 118 11–21. 10.1093/aob/mcw074 27208734 PMC4934396

[B82] Xie Z. Staehelin C. Vierheilig H. Wiemken A. Jabbouri S. Broughton W. J. (1995). Rhizobial nodulation factors stimulate mycorrhizal colonization of nodulating and non-nodulating soybeans. *Plant Physiol.* 108 1519–1525. 10.1104/pp.108.4.1519 12228558 PMC157531

